# Carrion in Bomas: Multiple Observations of Cheetah ( *Acinonyx jubatus* ) Scavenging Events and Potential Causes in Managed Populations

**DOI:** 10.1002/ece3.70776

**Published:** 2025-01-08

**Authors:** Elizabeth Kennedy Overton, Robert S. Davis, Franck Prugnolle, Virginie Rougeron, Terry‐Lee Honiball, Olivia Sievert, Jan A. Venter

**Affiliations:** ^1^ Department of Conservation Management Nelson Mandela University, George Campus George Western Cape South Africa; ^2^ International Research Laboratory REHABS, CNRS‐Université Lyon 1‐NMU, George Campus George South Africa; ^3^ Sustainability Research Unit Nelson Mandela University, George Campus George South Africa; ^4^ Endangered Wildlife Trust Midrand South Africa

**Keywords:** fenced reserves, Malawi, metapopulation, South Africa, translocations

## Abstract

Facultative scavenging can be observed across a large range of carnivorous mammals but is an uncommon behavioural trait in cheetahs ( *Acinonyx jubatus* ). Very few incidents of cheetahs scavenging have been reported, with no explanation given as to why it may occur. In this paper, we provide three more observations of cheetahs scavenging between 2019 and 2023 in three different protected areas in South Africa and Malawi. We suggest a common factor between these observations, namely that all adult individuals involved were temporarily housed in holding enclosures (bomas) during relocation processes and were provisioned with carrion as supplemental feed. The observed scavenging events could also have been influenced by the easy access to food in a situation where food acquisition was potentially difficult (i.e. old age, loss of hunting partner, mother with cubs). We hypothesise that these contributing factors, combined with the prior exposure of the adult individuals to eating carrion, could be a potential explanation for our observations of cheetahs scavenging. These observations provide a basis for further research into cheetah scavenging behaviour and the potential impacts of translocations that require prolonged holding periods on wildlife behaviour post‐release. Understanding these behavioural shifts is crucial for cheetah conservation, as successful reintroduction efforts depend on the ability of cheetahs to adapt to new environments and food acquisition strategies.

## Graphical abstract

Very few incidents of cheetahs ( *Acinonyx jubatus* ) scavenging have been reported, with no explanation given as to why it may occur. In this paper, we provide three observations of cheetahs scavenging and suggest a common factor between them: all adult individuals involved passed through bomas during relocation processes and were provisioned with carrion that they did not kill themselves.

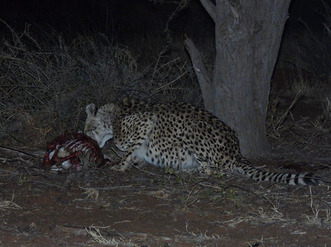

## 1 Introduction

Carnivores have developed different evolutionary strategies that allow them to maximise their energy intake required for survival (Ruxton and Houston 2004; Pereira, Owen‐Smith, and Moleón 2014; Skinner and Chimimba 2005), including predation, kleptoparasitism and scavenging (Allen, Elbroch, and Wittmer 2021; Inger et al. 2016; Schaller 1976). Scavenging can be defined as the act of feeding from the remains of dead animals that an individual did not kill itself (Schaller 1976), which can be advantageous when the provided overall energy intake is higher and the associated risks are lower than those linked to hunting prey (DeVault, Rhodes, and Shivik 2003). Scavengers are important ecological drivers because they limit carrion build‐up, reducing potential contamination and the spread of diseases (Le Sage, Towey, and Brunner 2019; Markandya et al. 2008; Ogada et al. 2012; Inger et al. 2016). There are two types of scavengers as defined by Beasley, Olson, and Devault (2015): obligate scavengers that rely on carrion for survival and reproduction (e.g. vultures, Ruxton and Houston 2004) and facultative scavengers that will scavenge but do not depend solely on this type of resource acquisition (e.g. jackals ( *Canis mesomelas* ), Hayward et al. 2017). The latter strategy can provide dietary flexibility and complementary resources that are both opportunistic and easily accessible (Pereira, Owen‐Smith, and Moleón 2014). However, scavenging can also represent risks for the consumer by increasing both the chance of predator encounters and infection risk (Honiball et al. 2024; Moleón et al. 2017; Moleón and Sánchez‐Zapata 2021; Périquet et al. 2021). An increased encounter rate between scavenging carnivores can heighten both the risk of conflict, particularly with more dominant species, and the transfer of pathogens (Moleón and Sánchez‐Zapata 2021). Scavengers can also be exposed to contaminants or diseases from the carcasses themselves, especially when the death of the prey is caused by potentially transmissible ailments (Jennelle et al. 2009; Legagneux et al. 2014). These risks associated with scavenging can therefore cause either injury or death to the consumer but can also facilitate the spread of pathogens throughout an ecosystem, which can be particularly harmful for species with reduced genetic diversity (Carrasco‐Garcia et al. 2018; Magliolo et al. 2023; Pienaar 1961).

Some species are selective when scavenging, appearing to avoid carrion under certain circumstances (e.g. visual cues to pathogen risk, several carcasses in a small space that could indicate pathogens or specific groups like carnivore carcasses, Moleón et al. 2017; Olson et al. 2022; Butler‐Valverde et al. 2022). Other carnivores tend to avoid scavenging altogether (e.g. African wild dogs ( *Lycaon pictus* ), cheetahs ( *Acinonyx jubatus* ), Hunter 1998; Sievert et al. 2023; Skinner and Chimimba 2005).

Cheetahs are a cryptic and skittish species that often live at low densities (average: 0.48/100 km^2^, Weise et al. 2017), resulting in many behavioural traits often going unnoticed or being scarcely reported (e.g. a new behaviour recorded by Davis et al. (2024) of cheetahs having kills stolen by baboons (*Papio sp*.)). Cheetahs' main foraging strategy is hunting, followed by gorging at a kill then moving off to digest in order to avoid competition with larger predators (i.e. leopard ( *Panthera pardus* ), spotted hyena ( *Crocuta crocuta* ) and lion ( *Panthera leo* ), Akbari et al. 2022; Scantlebury et al. 2014). Cheetahs rarely scavenge compared to other large carnivores, such as brown hyenas ( *Parahyaena brunnea* , Skinner and Chimimba 2005), avoiding the increased risk of encountering competing predators, which is one of their highest sources of mortality (Buk et al. 2018). The only times that cheetahs commonly eat prey that was not killed by themselves or a member of their group is when they are in captivity or small enclosures (also referred to as bomas), usually during management interventions such as relocations, rehabilitation or veterinary procedures (e.g. awaiting relocation or during recuperation periods, Houser 2008; Quirke, O'Riordan, and Zuur 2012). In these instances, cheetahs are usually fed fresh whole or sections of carcasses. This action of supplementary feeding has been found to influence the behaviour of captive cheetahs, causing them to sometimes show stereotypical behaviour (Quirke, O'Riordan, and Zuur 2012), consume the entirety of carcasses and remain with them for long periods of time or return to them over several days (Houser 2008). These signs have been found to diminish after release into larger enclosures with live prey. For example, during the rehabilitation of three young cheetahs in Botswana, Houser (2008) reported that only after two months post‐release in a 100 ha enclosure did the cheetahs start exhibiting a more natural feeding pattern on carcasses.

A few sporadic sightings of scavenging behaviour have been reported, but without providing any explanation as to why they occur. Pienaar (1969) reported observations of a cheetah scavenging in Kruger National Park, South Africa, on a buffalo ( *Syncerus caffer* ) killed by lions a few days prior. Two more reports of cheetahs scavenging on carcasses were documented in Etosha National Park, Namibia, and the Serengeti Plains, Tanzania (Caro 1982; Stander 1990). In Etosha, three cheetahs were observed feeding on a giraffe ( *Giraffa camelopardalis* ) carcass that was 1 day old, and in the Serengeti, a mother with her three cubs proceeded to eat a wildebeest ( *Connochaetes taurinus* ) carcass after unsuccessfully attempting to hunt within a large herd and scattering them. Broekhuis and Irungu (2016) even reported a record of a cheetah actively stealing a kill from a spotted hyena in the Maasai Mara National Reserve, Kenya. None of these individuals were reported as having undergone management interventions that require supplementary feeding (i.e. translocations).

In this paper, we describe new observations of cheetahs scavenging from carcasses. We suggest certain possible explanations regarding the development of this behaviour in these individuals that could be the basis for future studies on the impact of translocations on cheetahs and their surrounding environments (including behavioural ecology of cheetahs, predator guild dynamics, trophic interactions). All of the following scavenging events were carried out by cheetahs that have been relocated from one reserve to another. In the context of increasing management of cheetah populations, relocations are becoming a frequent strategy to mimic the natural dispersal of individuals when it is not possible (i.e. prohibited by fences, Magliolo et al. 2023). If pre‐exposure to carcasses during these procedures is in fact contributing to behaviour modifications in cheetahs, we suggest that there could be a potential increase in scavenging occurrences by cheetahs that have been in holding facilities.

## 2 Field Observations

Observations of cheetahs scavenging were obtained through direct observations and by‐catch data from carnivore research projects in three different locations: Tswalu Kalahari Reserve and Madikwe Game Reserve in South Africa and Liwonde National Park in Malawi. Details of the cheetahs involved, including birth dates, relocations and social structures, were obtained from the Cheetah Range Expansion Project for the Endangered Wildlife Trust (EWT).

### 2.1 Tswalu Kalahari Reserve

The Tswalu Kalahari Reserve is situated in the Northern Cape of South Africa and is over 1200 km^2^ in size. The main habitat types have been classified as duneveld, bushveld, calcrete scrubveld, shrubveld and mountain shrubveld. The area is split by electric fencing into two sections: the Greater Korannaberg (±900 km^2^) containing cheetahs, wild dogs, brown hyenas, spotted hyenas and leopards, and the Lekgaba section (±180 km^2^), home to lions, brown hyenas and leopards (pers. comm.). Due to this setup, cheetahs do not overlap with lions on the reserve. At the time of the following observation, the population of cheetahs was at a density higher than 1.22/100 km^2^ (equating the number of known adult individuals at the time), not including cubs or sub‐adult individuals of which there were several (pers. comm.).

**FIGURE 1:**
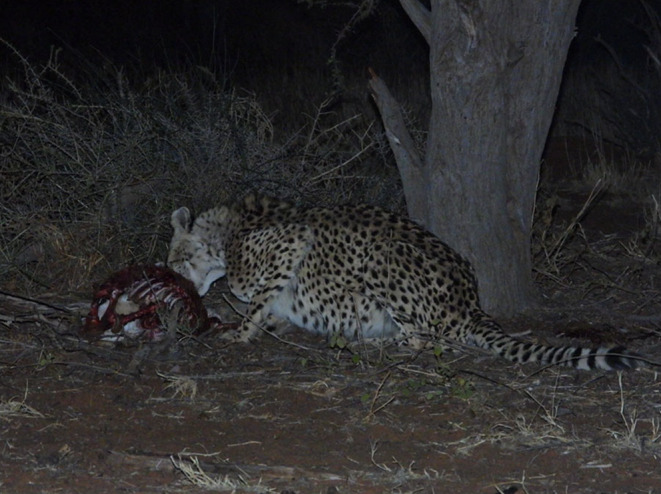
Photo of male cheetah at Tswalu Kalahari Reserve scavenging from springbok carcass. *Photo credit: Elizabeth Kennedy Overton*.

Direct observations of carnivores feeding on a springbok ( *Antidorcas marsupialis* ) carcass took place on 13 July 2023 at Tswalu Kalahari Reserve in an open space under a tree less than 100 m from a waterhole. A female cheetah with three cubs (approximately 4 months old) made the kill at around 12 h00 and the four individuals remained feeding until 17 h00. When the cheetahs left, several black‐backed jackals then scavenged from the remainder of the carcass until an old male cheetah (10 years old) appeared and approached the carcass. After several minutes of sniffing various contents on the ground, he proceeded to consume the remainder of the carcass (Figure 1). This male cheetah was wild born in October 2012 and relocated to Tswalu Kalahari Reserve from Phinda Private Game Reserve, South Africa in March 2014, spending time in holding bomas.

### 2.2 Madikwe Game Reserve

Madikwe Game Reserve is a 650 km^2^ fenced reserve in the North West province of South Africa, covered in Marico bushveld and Kalahari thornveld, ranging from dense to open habitat. According to intensive management and population estimates carried out by Honiball et al. (2021), carnivores in the reserve at the time included reintroduced lions (33 individuals), wild dogs (12 individuals), cheetahs (six individuals, density of 0.92/100 km^2^) and spotted hyenas (estimated 81 individuals) and naturally occurring brown hyenas (estimated 92 individuals) and leopards (estimated 24 individuals).

In Madikwe Game Reserve, five independent observations of a cheetah coalition comprising two males were observed at two separate baiting sites (sites 1 and 2, placed 100 m apart), situated in fairly open habitat. The baiting sites consisted of 50 kg of fresh impala ( *Aepyceros melampus* ) meat hoisted into trees for the purpose of leopard identification surveys (Honiball 2021). The baits were placed at both sites from 27 August 2019 until 17 September 2019. The cheetah coalition visited both sites on multiple occasions (as did other scavengers such as brown hyena, spotted hyena and black‐backed jackal). Observations were made on 29 August 2019 at 18 h22 (site 1) and 18 h43 (site 2), 11 September 2019 at 01 h56 (site 1) and 02 h37 until 02 h40 (site 2), and lastly 14 September 2019 at 23 h10 (site 2). Both males were also wild born at Phinda Private Game Reserve in July 2013 and were relocated on 29 November 2015 to Madikwe Game Reserve where they were released from holding after a period of minimum two weeks.

### 2.3 Liwonde National Park

Liwonde National Park is 548 km^2^ in size and is open to Mangochi Forest Reserve, adding on an additional 407 km^2^. Primarily consisting of mopane woodland, it is crossed by the Shire River that creates a system of floodplains. The density of cheetahs was of approximately 3.67/100 km^2^ (20 individuals) around the time of the following observation. Other carnivores included lions (~17 individuals) and spotted hyenas (~26 individuals), but no leopards occurred (pers. comm.).

**FIGURE 2:**
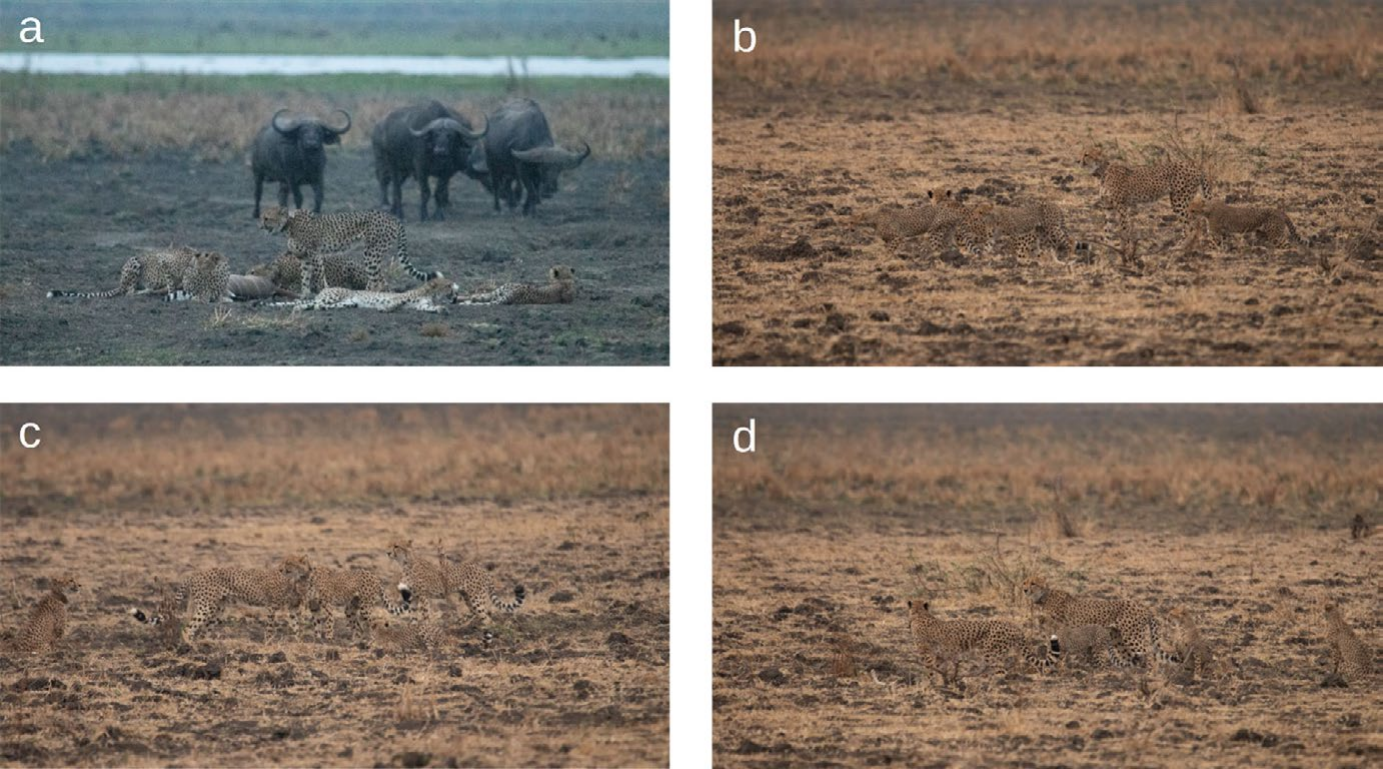
Photo sequence of (a) individuals from the cheetah group 1 (mother, three of her five sub‐adult cubs and two adult males) feeding from the kudu kill they made before the scavenging event and (b, c and d) individuals from both cheetah groups 1 and 2 (mother and cubs) interacting the following day (b–d). All adults are collared, the uncollared sub‐adults belong to the female from group 1 and the younger cubs whose manes are still visible belong to the female from group 2. *Photo credit: Benni Hintz and the Lilongwe Wildlife Trust Biodiversity Research team*.

In Liwonde National Park, a group of eight cheetahs consisting of an adult female, her five sub‐adult cubs and two adult males had killed a greater kudu ( *Tragelaphus strepsiceros* ) on 13 October 2021 (hereafter referred to as group 1, Figure 2a). While they were eating, a mother cheetah and her two sub‐adult cubs (group 2) approached and waited for group 1 to finish. Once they moved off the kill, group 2 approached the carcass and proceeded to eat what was unconsumed. All individuals were seen positively interacting at the site the following day (Figure 2b–d). The adult female cheetah in group 2 was relocated from Mountain Zebra National Park, South Africa, where she was wild born in September 2015. She was released into Liwonde National Park on 12 June 2017 and had undergone numerous months of holding prior to and one month after the international relocation.

## 3 Discussion

All three scavenging instances described here were carried out by cheetahs who were wild born but previously relocated at least once. These individuals were therefore kept for varying periods in holding enclosures, being fed carcasses they did not actively hunt. Supplementary feeding of carcasses or parts of carcasses to cheetahs in bomas could pre‐expose them to a behaviour that mimics scavenging in the wild, potentially reinforcing safety around this foraging strategy. This could make it more likely to happen and could be a potential explanation for our observations of cheetahs scavenging. However, it is interesting to note that individuals observed scavenging by Pienaar (1969), Caro (1982), Stander (1990) or Broekhuis and Irungu (2016) were never reported as having been translocated, held in bomas, nor provided with supplementary food.

Even for cheetahs who have been exposed to carcass supplementation in holding, they have been found to revert to hunting after release and scavenging does not remain their main source of food acquisition (Skinner and Chimimba 2005). It is important to point out that the scavenging events reported here could also potentially be influenced by other factors, including age, health or due to the presence of cubs. The male cheetah from Tswalu Kalahari Reserve was almost 11 years old and had lived in a coalition with his brother until the beginning of 2023 when the latter died (reported by management), months before the scavenging event took place. This change in group structure could have increased pressure on the individual to find food, as both age and sizes of male coalitions can affect their survival (Durant, Kelly, and Caro 2004). The female cheetah in Liwonde National Park was accompanied by her cubs, which also increases the pressure and frequency at which she must provide nourishment for herself and her young (Laurenson 1995; Hilborn 2018). The urgency to find food in this instance could outweigh the heightened risk of encountering competing predators when scavenging with her cubs. Madikwe Game Reserve has an intact large carnivore guild, placing pressure on subordinate predators such as cheetahs who have a high dietary overlap with lions, wild dogs and brown hyenas (Honiball et al. 2021) and who often lose their kills to kleptoparasitism. This high kleptoparasitic pressure could result in cheetahs needing to supplement their diet with carrion, particularly in fenced reserves where there is less available space for avoiding other predators, resulting in potentially higher food losses. As energy intake is one of the main factors defining resource acquisition strategies, the opportunistic carrion in all of these situations could have provided benefits that outweighed the risks associated with carrion avoidance (DeVault, Rhodes, and Shivik 2003). All of these events could therefore have been influenced by the easy access to food in a situation where food acquisition was potentially difficult. Observations of cheetahs returning to a kill or even attempting to cache food have also been made (personal observations by O.S., T.‐L.H and E.K.O. in all three parks where the scavenging events took place), however rare, reinforcing the argument for potential behaviour changes when there is an increased need for food. In two of the instances recorded in this paper, the cheetahs scavenged on animals that were killed by individuals of the same species less than 24 h prior. This could also potentially indicate safety of the carcasses to the individual scavenging after the departure of the cheetahs that made the kill.

If cheetahs who have been exposed to eating animals that they did not kill themselves are including occasional carrion into their diet, this could have varying consequences on their biology and the ecosystem around them. First, there is a risk of contamination if the scavenged carcass died from an illness or poison, or if the individual that killed it was ill (Ogada, Richards, and Behmke 2019). For example, reports have been made of cheetahs dying after eating zebra carcasses contaminated with anthrax in the Namib Desert during the 1960–61 epidemics and again in 2019 (Pienaar 1961; Portas et al. 2021). As the managed cheetah metapopulation approach expands through reintroductions to open areas or other countries, there could also be an increased risk of these types of contaminations, resulting in population establishment implications (Leighton et al. 2022; Marnewick et al. 2023; Ogada 2014; Ogada, Richards, and Behmke 2019). This can also particularly be a problem regarding the low genetic diversity of cheetahs (Magliolo et al. 2023), lessening the species' ability to cope with diseases (Spielman et al. 2004). Secondly, the encounter rates between cheetahs and more dominant predators around carcasses while scavenging could lead to injuries or death for the cheetah. Lastly, if cheetahs supplement their feeding habits with scavenging, this reduces their hunting impact on prey populations. In areas with high cheetah density, this could compensate for predatory competition and reduce pressure on prey populations. However, it could also increase competition between scavengers, influencing carnivore guild dynamics. If this behaviour is also passed onto offspring, whose prey preferences can resemble those of their mothers (Mills and Mills 2018), this could change the pressures that cheetahs exert on their surrounding ecosystems. If scavenging becomes a possibility for cheetahs after translocations or reintroductions, this could also lead to conflict with humans if they scavenge from large numbers of livestock (e.g. the reintroduction of 20 cheetahs to India, Marnewick et al. 2023).

Boma management needs to be focused on reducing the possibilities of causing changes in behaviour that could affect cheetah survival post‐release. Limiting the time a cheetah is placed within a boma could reduce its exposure to eating animals it did not kill itself. Removing carcasses after an individual has fed could prevent it from returning to them, which is not a common behaviour in the wild. Disposing of the removed carcasses far from the boma could reduce the possibility of other predators being lured to the boma. Without this incentive for scavengers to come near the boma, this might reduce the likelihood of the cheetahs inside becoming acclimatised to having other predators at close proximity.

This paper suggests that the exposure of cheetahs to eating carrion whilst in holding, combined with other factors that could reinforce the need for easy resources, could be a potential explanation for observations of them scavenging when released back into a natural habitat, even years later. This provides new opportunities for research to assess how relocations and feeding cheetahs in enclosures could modify their behaviour after release and, in turn, how this could influence ecosystem dynamics. Observations such as these are more commonly observed by people within reserves rather than during scientific studies and can therefore often be under‐represented in scientific literature. Another example of a species that is widely accepted as a non‐scavenger is the African wild dog, but people within reserves tend to be aware of scavenging tendencies post‐release from bomas (pers. comm.). This provides a good example of why observations outside of academic studies can be beneficial for uncovering natural history information.

## Author Contributions

**Elizabeth Kennedy Overton:** conceptualization (equal), data curation (lead), investigation (equal), visualization (lead), writing – original draft (lead), writing – review and editing (equal). **Robert S. Davis:** conceptualization (equal), data curation (equal), writing – review and editing (equal). **Franck Prugnolle:** writing – review and editing (equal). **Virginie Rougeron:** writing – review and editing (equal). **Terry‐Lee Honiball:** data curation (equal), writing – review and editing (equal). **Olivia Sievert:** conceptualization (equal), data curation (equal), writing – review and editing (equal). **Jan A. Venter:** writing – review and editing (equal).

## Conflicts of Interest

The authors declare no conflicts of interest.

## Data Availability

This article provides descriptive information so all available data are provided in photo format within the main document.
